# A hypermorphic antioxidant response element is associated with increased *MS4A6A* expression and Alzheimer's disease

**DOI:** 10.1016/j.redox.2017.10.018

**Published:** 2017-10-27

**Authors:** Sarah E. Lacher, Adnan Alazizi, Xuting Wang, Douglas A. Bell, Roger Pique-Regi, Francesca Luca, Matthew Slattery

**Affiliations:** a Department of Biomedical Sciences, University of Minnesota Medical School, Duluth, MN 55812, USA; b Developmental Biology Center, University of Minnesota, Minneapolis, MN 55455, USA; c Center for Molecular Medicine and Genetics, Wayne State University, Detroit, MI 48201, USA; d Environmental Epigenomics and Disease, Immunity, Inflammation and Disease Laboratory, National Institute of Environmental Health Sciences, National Institutes of Health, Research Triangle Park, NC 27709, USA; e Department of Obstetrics and Gynecology, Wayne State University, Detroit, MI 48201, USA

## Abstract

Late onset Alzheimer's disease (AD) is a multifactorial disorder, with AD risk influenced by both environmental and genetic factors. Recent genome-wide association studies (GWAS) have identified genetic loci associated with increased risk of developing AD. The *MS4A* (membrane-spanning 4-domains subfamily A) gene cluster is one of the most significant loci associated with AD risk, and *MS4A6A* expression is correlated with AD pathology. We identified a single nucleotide polymorphism, rs667897, at the *MS4A* locus that creates an antioxidant response element and links *MS4A6A* expression to the stress responsive Cap-n-Collar (CNC) transcription factors NRF1 (encoded by *NFE2L1*) and NRF2 (encoded by *NFE2L2*). The risk allele of rs667897 generates a strong CNC binding sequence that is activated by proteostatic stress in an NRF1-dependent manner, and is associated with increased expression of the gene *MS4A6A*. Together, these findings suggest that the cytoprotective CNC regulatory network aberrantly activates *MS4A6A* expression and increases AD risk in a subset of the population.

## 1 Introduction

Late-onset Alzheimer's disease (AD), which accounts for greater than 95% of all AD cases, has a complex multifactorial etiology. Neuroinflammation, proteostatic stress, oxidative stress, and mitochondrial dysfunction are all likely contributors to AD progression [1], [2], [3], [4], [5]. The various cellular stressors driving neurodegeneration in AD can be modified by genetic factors, and AD has a significant but complex heritable component [5], [6]. Recent genome-wide association studies (GWAS) have shed light on the genetic contributors to AD, identifying almost two-dozen loci associated with AD risk [7], [8], [9]. Nevertheless, how variation at these loci alters AD risk remains largely unclear. Because GWAS hits provide a window into the regulatory networks affecting disease risk, these variants represent high priority candidates for dissecting the molecular basis of AD.

The *MS4A* (membrane-spanning 4-domain family, subfamily A) gene cluster, located on chromosome 11q12, is one of the ten most significant AD-associated loci [7], [8], [9]. The *MS4A* genes encode highly similar four-transmembrane domain proteins, and recent work suggests these proteins act as chemosensors for a variety of exogenous and endogenous ligands, including fatty acids, peptides, and sulfated steroids [10]. Ligand binding by the MS4A proteins promotes calcium conductance, which likely plays a role in MS4A-mediated signaling, and these proteins might also act as adaptors for signal transduction complexes [10], [11], [12], [13]. The MS4A proteins are often expressed in subsets of myeloid or lymphoid cells, but expression is also observed outside of the hematopoietic system [11]. Consistent with strong expression in the hematopoietic system, both MS4A1 and MS4A2 are involved in immune cell function [11], [13], [14], [15], [16], [17], [18].

AD-associated single nucleotide polymorphisms (SNPs) in the *MS4A* cluster fall within a linkage disequilibrium (LD) block encompassing *MS4A2*, *MS4A3*, *MS4A4A*, *MS4A4E*, *MS4A6A* and *MS4A6E*, with strong evidence for an AD association at the *MS4A6A* locus [8]. The SNP rs610932, which is located in the 3’ untranslated region of *MS4A6A*, was first identified as AD-associated in a study based on individuals of European ancestry (odds ratio = 0.91; *P* = 1.2 × 10^−16^) [8], and the significance of this SNP has since been replicated in East Asian populations [19], [20], [21]. Rs610932 is also associated with atrophy rates in multiple AD-related brain structures [22], [23].

The mechanistic link between AD and the *MS4A* locus is unclear, but changes in *MS4A6A* expression are likely to be important. Associations with AD are evident when looking at *MS4A6A* expression levels: higher *MS4A6A* expression in brain tissue is associated with AD and with neuropathological measures of AD progression (Braak scores) [24]. A similar AD association is observed for *MS4A6A* expression in blood, with AD patients expressing higher levels of *MS4A6A* [25]. Importantly, there is also a genetic component to variation in *MS4A6A* expression in AD cohorts, where the risk (G) allele of rs610932 is associated with higher *MS4A6A* expression in blood. *MS4A6A* is expressed in monocytes, macrophages, and microglia, so it is likely that expression levels in whole blood are reflective of expression in innate immune cells of the central nervous system [25], [26], [27], [28].

The findings described above suggest a model in which a genetic variant at the *MS4A* cluster drives increased *MS4A6A* expression and AD susceptibility. Beyond that, however, details regarding the connection between *MS4A6A* variation and expression are lacking, in part because LD at the *MS4A* cluster makes it difficult to separate causal SNPs from nonfunctional, co-inherited SNPs. To address this, we integrated the *MS4A6A* GWAS results with multiple functional genomics datasets to identify a putative causal variant, rs667897, responsible for increased *MS4A6A* expression and AD risk. We then validated that the risk allele of this variant creates a functional antioxidant response element (ARE). This ARE is bound by the cytoprotective Cap-n-Collar (CNC) transcription factors NRF1 and NRF2, key regulators of the response to oxidative and proteostatic stress. Importantly, the ARE allele of rs667897 is associated with high *MS4A6A* expression, and this allele is strongly activated by proteostatic stress in an NRF1-dependent manner. Both oxidative and proteostatic stress are significant contributors to AD, and this work suggests that, in a subset of the population, the normal protective response to these stressors can adversely upregulate *MS4A6A* and increase risk of developing AD.

## 2 Results and discussion

### 2.1 A putative cis-regulatory SNP (cis-SNP) at the MS4A6A locus

*MA4A6A* expression levels are influenced by local genetic variation. In patients with AD, the risk (G) allele of rs610932 is associated with higher *MS4A6A* expression in blood [25]. Although this association was previously observed only in AD cohorts [25], data from the Genotype-Tissue Expression (GTEx) Consortium [29] indicate that a similar association is seen for rs610932 in non-AD populations (Table 1 and Fig. S1A). All major *MS4A6A* isoforms show a similar association with rs610932, suggesting that a variant linked to this SNP alters the activity of a *cis*-regulatory region (i.e., an enhancer) at the *MS4A6A* locus [25].

**Table 1:** Putative *cis*-regulatory variants in linkage disequilibrium with rs610932.

**SNP (rsID)**	***r***^**2**^**(relative to rs610932)**		**Effect on*****MS4A6A*****expression**#	
	EUR*	EAS**	**Effect size**	**P-value**
rs610932	1	1	0.13	1.2 × 10^−8^
rs667897	0.883	1	0.13	1.0 × 10^−8^
rs7933202	0.839	0.095	0.12	2.6 × 10^−6^

* EUR, European population.

** EAS, East Asian population.

# Data are from the Genotype-Tissue Expression (GTEx) Project.

**Fig. 1:**
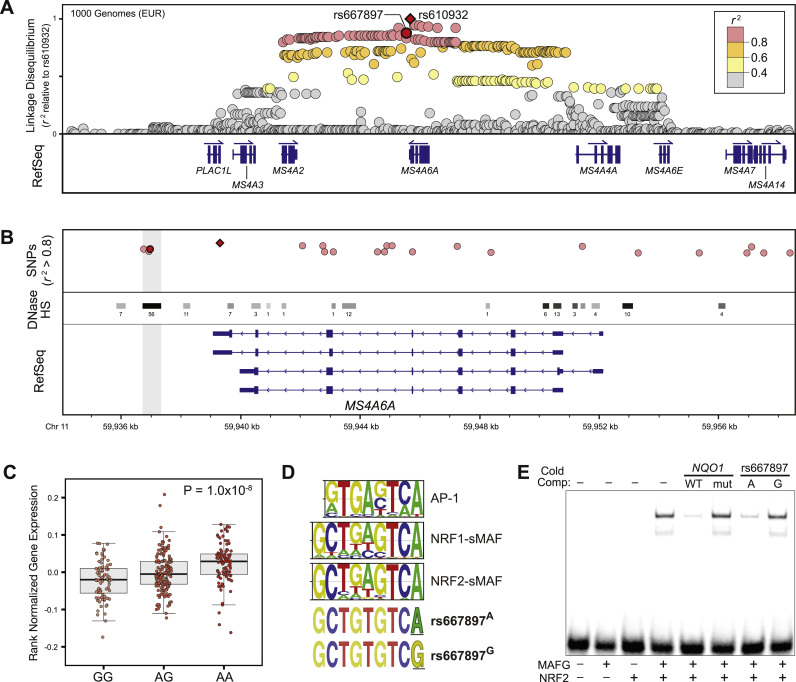
**A polymorphic antioxidant response element (ARE) at the*****MS4A6A*****locus. (A)** Linkage disequilibrium at the *MS4A* locus based on data from Phase 3 of the 1000 Genomes Project. All *r*^2^ values are relative to rs610932. **(B)** An expanded view of the *MS4A6A* locus. SNPs with *r*^2^ values >0.8 are highlighted in red. DNase hypersensitive sites (DHS), which represent putative regulatory DNA regions, are marked with black/grey bars. DHS data are summarized from 125 cell lines profiled by the ENCODE project [32]. The shading at each DHS is proportional to the strongest DNase signal in the ENCODE data, and the number associated with each DHS represents the number of cell lines in which that region is DNase hypersensitive. **(C)** Rank normalized *MS4A6A* expression levels across individuals, plotted by genotype at rs667897. The A allele is associated with increased *MS4A6A* expression in blood. Data are from the GTEx (Genotype Tissue Expression) project [29]. **(D)** Consensus motifs for AP-1, NRF1-sMAF, and NRF2-sMAF complexes aligned with the two alleles of rs667897. The A allele of rs667897 is a strong match to the CNC-sMAF motif (i.e. ARE) and AP-1 consensus motif. **(E)** NRF2-MAFG electrophoretic mobility shift assay (EMSA) using the canonical ARE from the *NQO1* promoter as a labeled probe. Lane 1 contains the labeled *NQO1* ARE probe with no protein, lane 2 contains the *NQO1* probe with purified MAFG only, lane 3 contains the *NQO1* probe with purified NRF2 only, and lane 4 contains the *NQO1* probe with NRF2 and MAFG. Binding is only seen when both proteins are present in the reaction. Lanes 5–8: NRF2 and MAFG are present in each lane. Competition reactions included addition of excess unlabeled competitor probes containing the wild-type (WT) *NQO1* ARE, a mutated (mut) *NQO1* ARE, the A allele rs667897, or the G allele of rs667897, as indicated. Competitor probes from the *NQO1* ARE and from the A allele of rs667897 compete for NRF2-MAFG binding to the labeled probe; mutated ARE probes and the G allele of rs667897 do not compete for binding. Abbreviations: *MS4A6A*, *membrane spanning 4-domains A, member 6 A*; DHS, DNase hypersensitive sites; AP-1, activator protein 1; NRF1, nuclear factor erythroid 2-related factor 1; NRF2, nuclear factor erythroid 2-related factor 1; sMAF, small MAF; CNC, cap-n-collar; MAFG, v-maf musculoaponeurotic fibrosarcoma oncogene family, protein G; *NQO1*, *NAD(P)H quinone dehydrogenase 1*.

To identify putative *cis*-SNPs contributing to differential expression of *MS4A6A*, we focused on variants in strong LD (r^2^ > 0.8) with rs610932 based on data from the 1000 Genomes Project [30]. We focused on European (EUR) and East Asian (EAS) populations because rs610932 is associated with AD in both populations [8], [19], [20], [21]. A total of 86 linked SNPs were identified for EUR, while EAS had 59 linked SNPs. We then used RegulomeDB [31] to search for SNPs likely to impact *cis*-regulatory activity. Specifically, we looked for SNPs that: (1) overlap a transcription factor (TF) binding site based on genome-wide chromatin immunoprecipitation (ChIP-seq) data, (2) overlap a TF binding site based on genome-wide DNase footprinting data, and (3) disrupt a putative TF-DNA binding sequence. This approach identified two putative *cis*-SNPs in EUR: rs667897 (r^2^ = 0.883) and rs7933202 (r^2^ = 0.839) (Fig. 1A-B and Table 1). In EAS, only rs667897 (r^2^ = 1.0) was identified as a putative *cis*-SNP; rs7933202 (r^2^ = 0.095) is not in LD with rs610932 in this population (Table 1). Like rs610932, both EUR SNPs are associated with *MS4A6A* expression levels in blood, although the association for rs667897 (P = 1.0 × 10^−8^) is somewhat stronger than rs7933202 (P = 2.6 × 10^−6^) (Table 1; see also Fig. 1C and Fig. S1A). Thus, relative to rs7933202, rs667897 is more strongly linked with the AD-associated SNP (rs610932) in both EUR and EAS populations, and it is strongly associated with *MS4A6A* expression levels. Overall these characteristics make rs667897 a high priority *cis*-SNP candidate, likely to have a functional impact on *MS4A6A* expression and, by extension, AD risk.

### 2.2 Allele-specific transcription factor binding at rs667897

The data implicating rs667897 as a functional *MS4A6A cis*-SNP also provide insight regarding the potential TFs acting at this SNP. Rs667897 is located in a putative enhancer region as indicated by DNase I hypersensitivity (Fig. 1B), and it falls within a DNase I footprint in this enhancer region [31], [32], [33], [34], [35]. DNase I footprints are small segments of uncleaved DNA within otherwise DNase hypersensitive regions; protection from DNase cleavage within the footprint is due to TF-DNA binding that prevents DNase from acting on a TF's DNA binding site [36]. The DNase I footprint encompassing rs667897 is centered on the sequence GCTGTGTC[A/G] – the polymorphic position, A or G, is in brackets. Importantly, the version of this sequence containing the A (risk) allele matches GCnnnnTCA, the consensus binding sequence for dimeric complexes of the Cap-n-Collar (CNC) and small MAF (sMAF) TFs. Specifically, this sequence matches the antioxidant response element (ARE) bound by complexes of NRF1 (encoded by *NFE2L1*) or NRF2 (encoded by *NFE2L2*), both of which are CNC factors, with one of the sMAF factors (MAFF, MAFG, or MAFK) (Fig. 1D) [37], [38], [39], [40], [41], [42], [43], [44]. The A allele sequence is also a single mismatch away from the consensus binding sequence (TGASTCA, where S = G or C) for dimeric AP-1 complexes, which consist of proteins from the FOS, JUN, and ATF subfamilies (Fig. 1D) [37], [38], [44], [45], [46], [47]. Additionally, ChIP-seq data from the ENCODE project demonstrate that the sMAF, FOS, and JUN proteins all bind to this enhancer region in vivo [33]. Thus, multiple lines of evidence suggest rs667897 falls within a DNA element bound by the CNC-sMAF and AP-1 regulatory complexes.

Both TF complexes implicated at rs667897 are responsive to stressors that have been linked to AD, and variation in their DNA binding sequences has been linked to heritable chronic disease risk [38], [48], [49], [50]. CNC-sMAF complexes regulate gene expression in response to proteostatic and/or oxidative stress, both of which are hallmarks of AD [1], [3], [4], [51], [52], [53]. Notably, activation of the CNC factors NRF1 and NRF2 is generally protective against neurodegenerative diseases including AD [3], [53], [54], [55]. AP-1 complexes are activated by many stimuli, including inflammatory cytokines and chemical stressors [56], [57]. AP-1 activity has never been linked directly to AD, but neuroinflammation is an important contributor to AD pathology [2], [58], [59]. Therefore, considering their biological functions, it is likely that disruption of a binding site for CNC-sMAF or AP-1 complexes at the *MS4A6A* locus could have implications for AD.

To determine whether CNC-sMAF or AP-1 complexes bind the sequence encompassing rs667897, we used electrophoretic mobility shift assays (EMSAs) to measure competition for binding with a labeled probe. We tested two CNC-sMAF complexes (NRF1-MAFG and NRF2-MAFG) and the canonical AP-1 complex (FOS-JUN), using a labeled DNA probe that is bound in vitro by all three complexes (Fig. 1E and Fig. S1B-C). The labeled probe is based on a CNC-sMAF binding site at the *NQO1* locus and, as expected, an unlabeled version of this probe efficiently competes for binding with the labeled probe. Mutation of the CNC-sMAF and AP-1 binding sequence (*NQO1*^mut^) eliminates competition for binding, demonstrating specificity of the assay for the CNC-sMAF and AP-1 complexes. Notably, competition with unlabeled probes from the sequence encompassing rs667897 revealed a marked allele-specific pattern. For all three protein complexes, the A allele of rs667897 efficiently competes for binding with the labeled probe, whereas the G allele does not compete (Fig. 1E and Fig. S1B-C). Importantly, this is consistent with patterns within DNase footprinting data, where the A allele is associated with a significantly stronger signal (i.e., stronger TF binding) than the G allele (allelic imbalance q-value = 1.74 × 10^−07^, see [60]). Thus, rs667897^A^, which is associated with increased *MS4A6A* levels and increased risk of AD, creates binding site for the CNC-sMAF and AP-1 complexes, and this binding site is eliminated by rs667897^G^.

### 2.3 Proteostatic stress activates rs667897^A^ in an NRF1-dependent manner

**Fig. 2:**
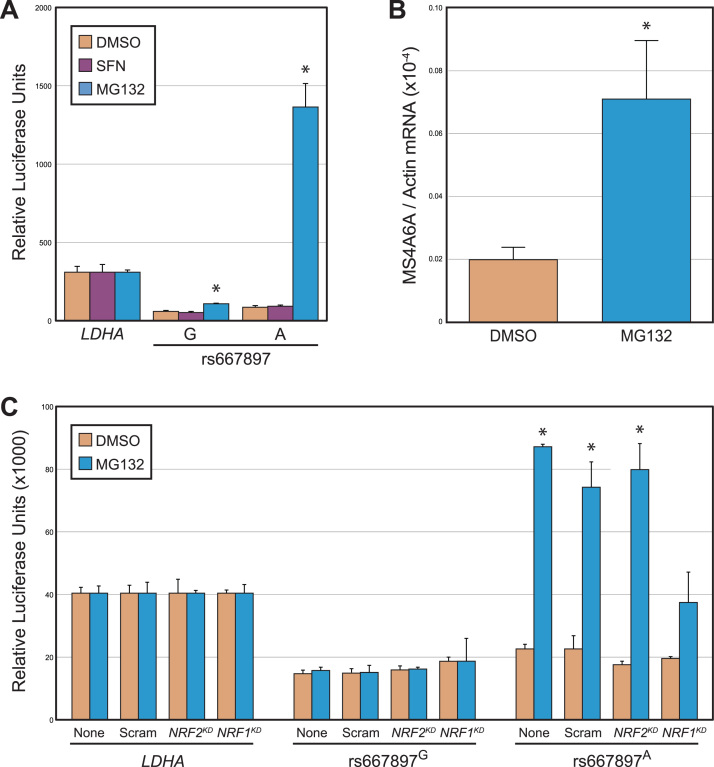
**The ARE allele of rs667897 is responsive to NRF1 activation. (A)** Allele-specific reporter assays in which the region encompassing rs667897^G^ (non-ARE allele) or rs667897^A^ (ARE allele) was cloned upstream of luciferase and transfected into HepG2 cells. Luciferase driven by the *LDHA* promoter was included as an internal control. Transfected cells were treated with vehicle (DMSO) control, sulforaphane, or MG132. **(B)** Quantitative, reverse transcription PCR monitoring of MG132-responsive *MS4A6A* expression in peripheral blood monocytes heterozygous (A/G) at rs667897. *MS4A6A* expression is significantly increased in MG132-treated cells. **(C)** Allele-specific HepG2 reporter assays, using the same rs667897^G^ (non-ARE allele) or rs667897^A^ (ARE allele) constructs from (A), only with knockdown of *NRF1* (*NRF1*^KD^) or *NRF2* (*NRF2*^KD^) using RNA interference. Cells were also treated with no RNAi (“none”) or scrambled RNAi duplexes (“Scram”) as negative controls. For all panels, values represent mean ± standard deviation, treatments were compared to the corresponding DMSO control, and asterisks represent an FDR-adjusted p-value < 0.05 (Welch's *t*-test).

To further explore the *cis*-regulatory activity of the two rs667897 variants, we generated allele-specific reporter constructs containing the CNC-sMAF and AP-1 binding site driving expression of the luciferase reporter gene. We then tested the activity of these reporter constructs in HepG2 cells – a cell type with high transfection efficiency and evidence for AP-1, NRF1-sMAF, and NRF2-sMAF activity [43], [61], [62] – in the presence or absence of chemical activators of each TF complex. Specifically, we measured reporter activity in response to: (1) sulforaphane (SFN), an activator of NRF2-sMAF activity; (2) MG132 a proteasome inhibitor that activates both NRF1-sMAF and NRF2-sMAF activity; or (3) phorbol myristate acetate (PMA), an activator of AP-1 activity (Fig. 2A and Fig. S2A) [63], [64], [65], [66], [67], [68]. Neither construct was upregulated by PMA or SFN. However, MG132 treatment resulted in strong upregulation of the rs667897^A^ construct, (16.6-fold induction), but little change in the rs667897^G^ construct (2.1-fold induction). Thus activators of AP-1 and NRF2 have little impact on the *cis*-regulatory output of the region encompassing rs667897, at least in HepG2 cells, whereas disruption of proteostasis with MG132 clearly affects rs667897's *cis*-regulatory activity.

Reporter assays in a highly transfectable cell line are an ideal system for allele-specific enhancer dissection experiments, but we also wanted to confirm that the *MS4A6A* transcript is responsive to MG132. The entire *MS4A* gene cluster is epigenetically silenced in most immortalized or cancer cell lines ([69], [70] and data not shown), so *MS4A6A* expression could not be tested in HepG2 or other standard cell lines. However *MS4A6A* is expressed in cells of the monocyte lineage, so we tested its response to MG132 in primary peripheral blood monocytes (PBMCs) heterozygous at rs667897. Indeed, *MS4A6A* expression is significantly induced by MG132 in PBMCs (Fig. 2B). Overall, in combination with the reporter assay data, these results indicate that *MS4A6A* is activated by disruption of proteostasis with MG132, and the A allele of rs667897 is significantly more responsive than the G allele to this proteostatic stress.

The proteostatic stress-responsive A allele of rs667897 creates a binding site for NRF1-sMAF or NRF2-sMAF, both of which are activated by MG132. To further dissect the precise molecular mechanisms at this *cis*-SNP, we next asked which TF is responsible for allele-specific activation of rs667897^A^. Overexpression of NRF1 or NRF2 resulted in allele-specific activation of the rs667897^A^ construct (Fig. S2B), demonstrating that an increase in either NRF1 or NRF2 is sufficient for induction of rs667897^A^ *cis*-regulatory activity. We then used siRNA knockdown to test whether NRF1 or NRF2 is necessary for MG132-mediated induction of rs667897 (Fig. 2C). Consistent with results from Fig. 2A, the rs667897^G^ construct did not respond to MG132, so knockdown of NRF1 or NRF2 had no effect on this allele. In contrast, the rs667897^A^ construct was induced by MG132, and this induction decreased significantly with knockdown of NRF1 (Fig. 2C and Fig. S3C). Unlike NRF1, knockdown of NRF2 had no effect on the induction of rs667897^A^ by MG132. NRF2 RNAi did decrease activity of the canonical *NQO1* ARE (not shown) so this negative result is not due to inefficient NRF2 knockdown. Thus, both NRF1 and NRF2 have the potential to upregulate *cis*-regulatory activity at rs667897^A^, but only NRF1 is necessary for full activation of this *cis*-SNP in response to MG132. Together these results demonstrate a central role for NRF1 in regulating expression of *MS4A6A* via the rs667897^A^ *cis*-SNP.

## 3 Conclusion

Proteostatic stress is an early and ongoing contributor to multiple neurodegenerative diseases, including AD [1], [3], [4]. Our data suggest that a protective response to proteostatic stress, directed by the NRF1-sMAF complex, actually promotes *MS4A6A* overexpression and AD risk in people carrying the risk (A) allele of rs667897. NRF2-sMAF and AP-1 bind the rs667897^A^ AD risk allele as well, so it is possible that these complexes also drive stress-responsive overexpression of *MS4A6A* in environments not covered by the assays described here. It should also be noted that both NRF1 and NRF2 can partner with individual AP-1 factors to regulate ARE-driven gene expression [71], [72], [73]; thus, the combinatorial TF possibilities at rs667897^A^ are numerous. In addition, the activities of NRF1 and NRF2 are closely linked, in part because proteostatic stress also disrupts redox homeostasis [52]. Nevertheless, although it is possible that the AP-1 factors and NRF2 might ultimately regulate *MS4A6A* in certain contexts, the work described here provides evidence for an NRF1-mediated mechanism by which genetic variation at the *MS4A* locus modulates expression of *MS4A6A*.

In the central nervous system *MS4A6A* is primarily expressed in cells of the monocyte/microglia lineage [28], [74]. Proteostatic stress and protein aggregation is associated with microglial activation and neuroinflammation in multiple neurodegenerative diseases, including AD [2], [75]. Thus, in the context of rs667897^A^, *MS4A6A*'s stress-responsive regulation and cell-specific expression are spatiotemporally relevant to AD. Importantly, because the activity of rs667897^A^ is modulated by stress, the significance of its association with AD could be modified by unlinked genetic variants or environmental exposures. Clearly future work will be necessary to uncover potential gene-gene or gene-environment interactions with *MS4A6A*, and to determine the precise role of *MS4A6A* in AD. Regardless, the characteristics described above put it in an interesting position that could bridge proteostatic disruption, neuroinflammation, and AD progression.

## 4 Materials and methods

### 4.1 Genomic data analysis

The LDproxy tool within the LDlink suite [76] was used to identify SNPs in linkage disequilibrium (LD) with rs610932; all analyses were based on data from Phase 3 of the 1000 Genomes Project [30]. SNPs in LD with rs610932 were then filtered using RegulomeDB [31] to identify polymorphisms most likely to disrupt transcription factor binding sites; SNPs with a RegulomeDB score of 1a, 1b, 2a, or 2b were considered putative *cis*-regulatory SNPs. Clustered DNase hypersensitive sites used in Fig. 1, based on data from the ENCODE project [32], were downloaded from the UCSC Genome browser at http://hgdownload.cse.ucsc.edu/goldenPath/hg19/encodeDCC/wgEncodeRegDnaseClustered/ (file name: wgEncodeRegDnaseClusteredV3.bed.gz). Processed genotype versus *MS4A6A* expression data (Fig. 1C and Fig. S1A) were obtained from the GTEx Portal (V6 data; https://www.gtexportal.org/home/) [29]. The GTEx Project was supported by the Common Fund of the Office of the Director of the National Institutes of Health, and by NCI, NHGRI, NHLBI, NIDA, NIMH, and NINDS.

### 4.2 Chemicals and reagents

D,L-sulforaphane (SFN) was purchased from MP Biomedicals LLC (Santa Ana, CA). MG132 was purchased from Sigma-Aldrich, (St. Louis, MO). Stock solutions of PMA, SFN, and MG132 test compounds were prepared in dimethyl sulfoxide (DMSO) and stored at −20 °C. FuGENE HD transfection reagent was purchased from Promega (Madison, WI). DharmaFECT Duo transfection reagent was purchased from Dharmacon (Lafayette, CO). His-tagged NRF2 protein was purified from BL21 bacteria transfected with pPROHEX-HC-Flag3-NRF2 as His-tagged fusions using Ni-agarose beads as described previously [77], [78]. NRF1 protein was generated using the TNT Quick Coupled Transcription/Translation System (Promega) programmed with the pPB-N-His-NRF1 plasmid from abm (catalog number PV028854; Vancouver, Canada). Purified FOS and JUN proteins were purchased from ProteinOne (Rockville, MD) (catalog numbers P2013-01 and P2014-01, respectively). Purified MAFG was purchased from ATGen Co. Ltd. (Los Angeles, CA). MISSION LightSwitch Luciferase Assay Reagent was purchased from Sigma-Aldrich. The NRF2 expression vector used in reporter assays, pCDNA3-Myc3-NRF2, was a gift from Yue Xiong (Addgene plasmid #21555) [79]. The NRF1 expression vector used in reporter assays, pcDNA3.1-NRF1-3xFlag, was kindly provided by Ray Deshaies [66]. All oligonucleotides used for electrophoretic mobility shift assays and cloning were purchased from Integrated DNA Technologies (Coralville, IA). siRNA and shRNA constructs were purchased from Santa Cruz Biotechnology (Santa Cruz, CA).

### 4.3 Electrophoretic mobility shift assays (EMSAs)

Human purified His-tagged NRF2 (75 nM), MAFG (37.5 nM), FOS (75 nM), JUN (75 nM), or TNT- prepared human NRF1 (2μl/20μl reaction), and IRDye-700 labeled double stranded DNA were incubated for 30 min at room temperature with poly(dI-dC) in the binding buffer (20 mM HEPES, 4 mM MgCl_2_, 100 μg/ml BSA, 4% glycerol, 20 mM KCl, 5 mM DTT, and 1 mM EDTA). Orange loading dye (LI-COR, Lincoln, NE) was added and samples were electrophoresed through a native acrylamide gel in 1X TBE. Gels were imaged using the Odyssey infrared imaging system (LI-COR, Lincoln, NE). Double stranded oligonucleotides containing the following sequences were used EMSA: 5’-AATCCGCAGTCACA**GTGACTCAGCA**GAATCTGAGCCTAG-3’ (wild-type *NQO1* ARE, *NQO1*^WT^); 5'-AATCCGCAGTCACA**GACTCCTACGA**GAATCTGAGCCTAG-3’ (mutant *NQO1* ARE, *NQO1*^mut^), 5’-CTGCGCTCCAAACC**CGCTGTGTCAT**ACCATACCGGATGT-3’ (*MS4A6A* ARE, rs667897^A^ allele), 5’-CTGCGCTCCAAACC**CGCTGTGTCGT**ACCATACCGGATGT-3’ (*MS4A6A* ARE, rs667897^G^ allele).

### 4.4 Cell culture

The human hepatocellular carcinoma HepG2 cells and peripheral blood mononuclear cells (PBMCs, Lot #700311) were purchased from the American Type Culture Collection (ATCC; Manassas, VA). HepG2 cells were grown and maintained in EMEM culture media (ATCC), supplemented with 10% fetal bovine serum purchased from Atlanta Biologicals (Norcross, GA), and 1% (v/v) penicillin/streptomycin purchased from Invitrogen-Life Technologies (Carlsbad, CA), and gown at 37 °C in humidified 5% CO_2_ air. PBMCs were grown in RPMI 1640 (ATCC) supplemented with 3 μg/ml Lectin from *Phaseolus vulgaris* (PHA, Sigma-Aldrich), 10% heat-inactivated fetal bovine serum, and 1% (v/v) penicillin/streptomycin, and gown at 37 °C in humidified 5% CO_2_ air. PBMC Lot #700311 was genotyped by Sanger sequencing using primers flanking rs667897: 5’-GGCTTTATTCTCTCTCCTGTCC-3’ (forward primer) and 5’-CACTTGCTACCTTTGTCCTTTAC-3’ (reverse primer).

### 4.5 Reporter gene assays

All reporter plasmids made use of the Switchgear Genomics (Carlsbad, CA) LightSwitch optimized luciferase reporter vector system. The *LDHA* (*lactate dehydrogenase A*) control reporter construct was purchased directly from Switchgear Genomics (Product ID: S721613). To generate the *MS4A6A* constructs (encompassing rs667897^A^ or rs667897^G^), we annealed oligonucleotides containing the ARE enhancer sequences into the pLightSwitch-LR Reporter Vector (SwitchGear Genomics, catalog number 32024). The pLightSwitch vector was cut with BglII and MluI purchased from New England Biolabs (Ipswich, MA), and the annealed oligonucleotides containing overhanging sequences compatible with a BglII and MluI insertion site. Annealing the following two oligonucleotides generated the rs667897^A^ insert: 5’-*CGCG*AAACCCGCTGTGTC**A**TACCAT-3’ and 5’-*GATC*ATGGTA**T**GACACAGCGGGTTT-3’ (ARE region is underlined; rs667897^A^ position is bold; and overhang cloning sequences are italicized). An equivalent oligonucleotide pair generated the rs667897^G^ insert: 5’-*CGCG*AAACCCGCTGTGTC**G**TACCAT-3’ and 5’-*GATC*ATGGTA**C**GACACAGCGGGTTT-3’. For reporter assays, cells were seeded and transfected in Opti-MEM (Invitrogen) supplemented with 10% FBS. 96 well plates were seeded with 15,000 cells/well and simultaneously transfected using the SwitchGear Genomic High-throughput transfection protocol. Each transfection included 0.15 μL of FuGENE HD transfection reagent and 50 ng/well of reporter plasmid. Additionally, some experiments were carried out in which either the pCDNA3-vector, pCDNA3-Myc3-NRF2, or pCDNA3-NRF1 plasmids were also co-transfected with the reporter plasmids at 2.5 ng/well. Other experiments were carried out in which either scrambled siRNA, *NRF2* siRNA, or *NRF1* siRNA were cotransfected with the reporter constructs. In this case, each transfection included 0.15 μL of DharmaFECT Duo transfection reagent, approximately 100 ng of reporter plasmid and 50 nM of siRNA. The experimental set up employed when DMSO, SFN, PMA, or MG132 were utilized is as follows: each construct was transfected in triplicate or quadruplicate and plates were incubated at 37 °C. The transfected cells were treated with DMSO, SFN, PMA, or MG132 24 h post transfection. Cells were then incubated for an additional 24 h at 37 °C before being frozen at −80 °C. The experimental set up employed when no chemical induction was utilized is as follows: each construct was transfected in triplicate and plates were incubated at 37 °C for 24 h, after which cells were frozen at −80 °C. Plates were removed from the freezer and allowed to reach room temperature. MISSION LightSwitch Luciferase Assay Reagent (100 μL) was added to each well, and plates were incubated at room temperature for 30 min and read on a SpectraMax M3 (Molecular Devices, Sunnyvale, CA) according to the manufacturers instructions.

### 4.6 Quantitative reverse transcription PCR

PBMCs were seeded at 1.5 million cells/ml in 100 mm petri dish and grown at 37 °C for 24 h (supplemented with 3 μg/ml PHA). Following which cells were treated with either vehicle (0.1% DMSO), or 2 μM MG132 24 h. DMSO concentrations remained at a constant concentration of 0.1% across all treatment groups. Following drug treatment for 24 h, total RNA was isolated using the Qiagen RNeasy kit. Reverse transcription was performed according to manufacturer's instructions (Omniscript RT kit, Qiagen) using 1.0 μg of total RNA to generate cDNA. Primers for *MS4A6A* (FAM-hydrolysis probe), *β-Actin* (gene symbol *ACTB*) (HEX-hydrolysis probe), and *TBP* (HEX-hydrolysis probe) were purchased from the predesigned PrimeTime qPCR Assays, Integrated DNA Technologies (Assay name: Hs. PT.58.28269192, probe based primers only, spanning exons 4–6). Integrated DNA Technologies designed gblocks as standards that contained sequences specific to the PrimeTime primers utilized. RT-PCR reactions were performed using a LightCycler 480 Multiwell Plate 96 containing 5 μM of each primer set, 1x LightCycler 480 Probes Master (Roche Diagnostics, Bazel, Switzerland), and 2μl of cDNA template, in a final reaction volume of 10μl. The RT-PCR was performed using the following cycle parameters: initial enzyme activation at 95 °C for 10 min; followed by 45 cycles of 95 °C for 10 s, 60 °C for 20 s, and 65 °C for 30 s. Following the amplification phase, a cooling step was performed at 40 °C for 10 s (ramp rate of 1.5 °C/s). Acquisition of the fluorescence signal was performed using the Dual Hydrolysis Probe setting for FAM (465–510 nm) and HEX/Yellow555 (533–580 nm) dyes following the 65 °C extension phase of each cycle. Relative expression of selected genes was evaluated using qPCR by calculating the ratio of the specific gene to the housekeeping genes *β-Actin* or *TBP*.

### 4.7 Statistical analysis

Statistical analysis was performed using Graphpad Prism (La Jolla, CA); statistical significance (Welch's *t*-test) was accepted at p < 0.05.
