# Supplementary material for: A hypermorphic antioxidant response element is associated with increased MS4A6A expression and Alzheimer's disease
Source: Redox Biol. 2017 Oct 27;14:686–93. doi: 10.1016/j.redox.2017.10.018 (PMC5705802; doi:10.1016/j.redox.2017.10.018)
Supplement: Supplementary file 1 — Supplementary material [file mmc1.pdf]

SUPPLEMENTARY FIGURES

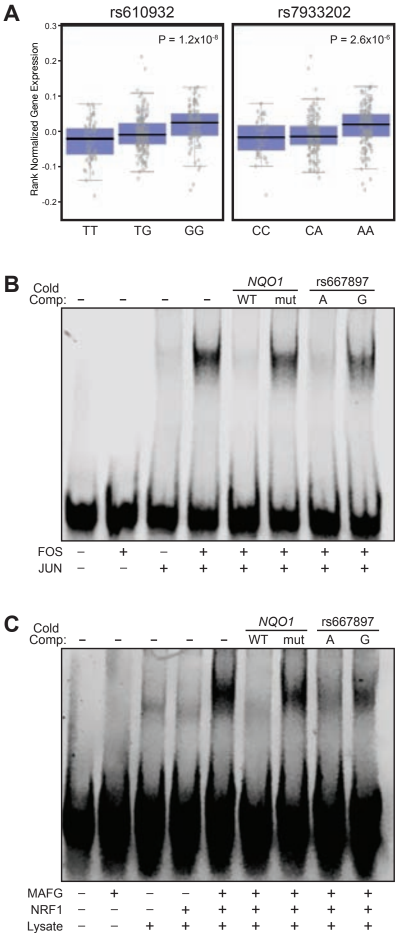

Figure S1

(A) Rank normalized *MS4A6A* expression levels across individuals, plotted by genotype at rs610932 (left panel) or rs7933202 (right panel). Data are from the GTEx (Genotype Tissue Expression) project.

(B) FOS-JUN electrophoretic mobility shift assay (EMSA) using the canonical ARE from the *NQO1* promoter as a labeled probe. Lane 1 contains the labeled *NQO1* ARE probe with no protein, lane 2 contains the *NQO1* probe with purified FOS only, lane 3 contains the *NQO1* probe with purified JUN only, and lane 4 contains the *NQO1* probe with FOS and JUN. Binding is only seen when both proteins are present in the reaction. Lanes 5-8: FOS and JUN are present in each lane. Competition reactions included addition of unlabeled competitor probes containing the wild-type (WT) *NQO1* ARE, a mutated (mut) *NQO1* ARE, the A allele rs667897, and the G allele of rs667897. Competitor probes from the *NQO1* ARE and from the A allele of rs667897 compete for FOS-JUN binding to the labeled probe; mutated ARE probes and the G allele of rs667897 do not compete for binding.

(C) NRF1-MAFG electrophoretic mobility shift assay (EMSA) using the canonical ARE from the *NQO1* promoter as a labeled probe. This is similar to EMSAs in Figure 1E and Figure S1B, although NRF1 protein was generated using a cell free expression system (see Methods). Lane 1 contains the labeled *NQO1* ARE probe with no protein, lane 2 contains the *NQO1* probe with purified MAFG only, lane 3 contains the *NQO1* probe with the unprogrammed cell free expression lysate (control lane, no NRF1 expressed), lane 4 contains the *NQO1* probe with lysate expressing NRF1 protein, and lane 5 contains the *NQO1* probe with both NRF1 and MAFG. Binding is only seen when both proteins are present in the reaction. Lanes 6-9: NRF2 and MAFG are present in each lane. Competition reactions included addition of unlabeled competitor probes containing the wild-type (WT) *NQO1* ARE, a mutated (mut) *NQO1* ARE, the A allele rs667897, and the G allele of rs667897. Competitor probes from the *NQO1* ARE and from the A allele of rs667897 compete for NRF1-MAFG binding to the labeled probe; the mutated ARE probe does not compete for binding, and the G allele of rs667897 does not compete as efficiently as the A allele of rs667897.

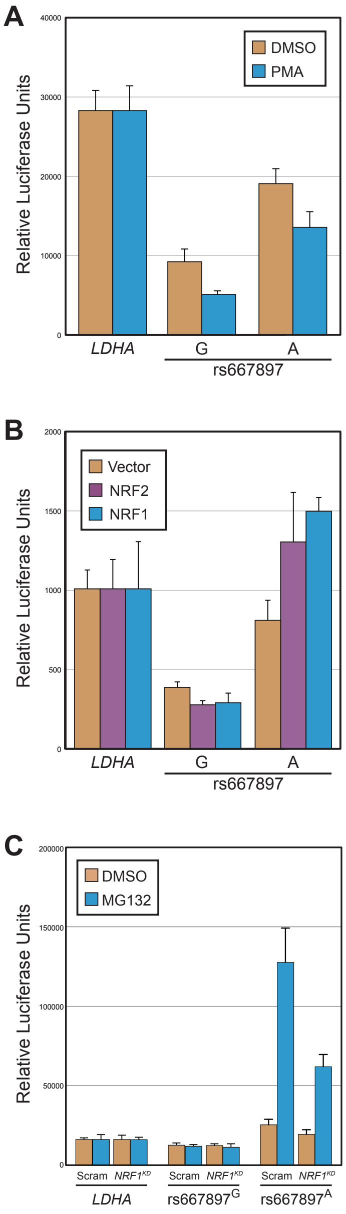

Figure S2

(A) Allele-specific reporter assays in which the region encompassing rs667897^{G} (non-ARE allele) or rs667897^{A} (ARE allele) was cloned upstream of luciferase and transfected into HepG2 cells. Luciferase driven by the *LDHA* promoter was included as an internal control. Transfected cells were treated with vehicle (DMSO) control or 100 nM PMA. Neither construct is activated by PMA. Similar results were seen with 500 nM PMA (not shown).

(B) Allele-specific reporter assays using luciferase reporter constructs described in (A). Reporter constructs were co-transfected into HepG2 cells with a control vector (pCDNA3) or pCDNA3-based NRF2 and NRF1 overexpression constructs.

(C) Same as (A), only with shRNA-mediated knockdown of *NRF1* ("*NRF1*^{KD}"). Reporter constructs were co-transfected into HepG2 cells with constructs expressing a control, scrambled shRNA ("Scram") or an shRNA targeting ("*NRF1*^{KD}"). As in Figure 1C, knockdown of *NRF1* inhibited MG132-mediated activation of rs667897^{A}.
